# Intergenerational Mentoring and Social Support: Evidence from the BRIDGE2Health Program

**DOI:** 10.1093/geroni/igaf122.3298

**Published:** 2025-12-31

**Authors:** Jill Juris, Shannon Jarrott, Hannah D’Angelo, Calyope Ortega

**Affiliations:** Appalachian State University, Boone, North Carolina, United States; The Ohio State University, Columbus, Ohio, United States; Appalachian State University, Boone, North Carolina, United States; Appalachian State University, Boone, North Carolina, United States

## Abstract

Social connections contribute to healthy aging. Intergenerational strategies provide an innovative approach to creating opportunities for meaningful social connections. BRIDGE2Health is a shared mentoring program designed to improve intergenerational social connections. Two cohorts of adults (n = 58, age range = 50-79) participated in monthly intergenerational programming with teens in one of two communities. The Interpersonal Support Evaluation List (Cohen et al., 1985), was utilized to measure functional social support. Paired samples T-Tests results indicated significant differences between pre and post-measures in three domains of functional support: appraisal (p < .001), tangible (p < .001), and belonging (p < .001) for adults in both communities. When comparing new (one year of participation) and returning participants (two years of participation), both new and returning participants experienced similar levels of change in social connection. For returning participants, the effect size was greater in appraisal (d = -2.17) compared to overall findings (d = -2). For new participants, belonging had the strongest difference of the three (appraisal, belonging, tangible). This study illustrates the potential for enhancing social connections through intergenerational programming.

